# Humin Assists Reductive Acetogenesis in Absence of Other External Electron Donor

**DOI:** 10.3390/ijerph17124211

**Published:** 2020-06-12

**Authors:** Mahasweta Laskar, Takuya Kasai, Takanori Awata, Arata Katayama

**Affiliations:** 1 Graduate School of Engineering, Nagoya University, Nagoya 464-8603, Japan; laskar.mahasweta@k.mbox.nagoya-u.ac.jp (M.L.); kasai.takuya@imass.nagoya-u.ac.jp (T.K.); 2 Institute of Materials and Systems for Sustainability, Nagoya University, Nagoya 464-8603, Japan; 3 National Institute for Land and Infrastructure Management, Tsukuba 305-0804, Japan; awata-t92yu@mlit.go.jp

**Keywords:** humin, reductive acetogenesis, CO_2_ reduction, autotrophic, dechlorination, methanogenesis

## Abstract

The utilization of extracellular electron transfer by microorganism is highly engaging for remediation of toxic pollutants under “energy-starved” conditions. Humin, an organo-mineral complex of soil, has been instrumental as an external electron mediator for suitable electron donors in the remediative works of reductive dehalogenation, denitrification, and so forth. Here, we report, for the first time, that humin assists microbial acetogenesis as the extracellular electron donor using the electron acceptor ${\mathrm{CO}}_{2}$. Humin was obtained from Kamajima paddy soil, Japan. The anaerobic acetogenic consortium in mineral medium containing ${\mathrm{CO}}_{2}/{\mathrm{HCO}}_{3}^{-}$ as the inorganic carbon source used suspended humin as the energy source under mesophilic dark conditions. Retardation of acetogenesis under the ${\mathrm{CO}}_{2}$-deficient conditions demonstrated that humin did not function as the organic carbon source but as electron donor in the ${\mathrm{CO}}_{2}$-reducing acetogenesis. The consortium with humin also achieved anaerobic dechlorination with limited methanogenic activity. Total electron-donating capacity of humin was estimated at about 87 µeeq/g-humin. The metagenomic sequencing of 16S rRNA genes showed the predominance of *Firmicutes* (71.8 ± 2.5%) in the consortium, and *Lachnospiraceae* and *Ruminococcaceae* were considered as the ${\mathrm{CO}}_{2}$-reducing acetogens in the consortium. Thus, microbial fixation of ${\mathrm{CO}}_{2}$ using humin introduces new insight to the holistic approach for sustainable treatment of contaminants in environment.

## 1. Introduction

The anaerobic nonphototrophic CO_2_ fixers are abundant in nature [1,2], and though energetics of CO_2_ reduction to acetate is less favorable than methanogenesis, the ability to use diverse electron donors sets the acetogens apart from the other microbial populations [3,4]. From the variability in chemolithotrophic acetogenesis [5]; to electron harvest using electricity [6]; to higher biochemical efficiency during CO_2_ fixation [7], reductive acetogenesis attracted much attention, particularly for the autotrophic Wood–Ljungdahl pathway (WLP) in acetate production ($2{\mathrm{CO}}_{2}+4H_{2}\to {\mathrm{CH}}_{3}\mathrm{COOH}+2H_{2}O)$ [8], although the reaction requires highly reduced conditions −290mV [1] as standard redox potential at pH 7 ($E^{o^{\prime }}$). In dark subsurface environments where organic substrates and energy are limited, acetate produced by the autotrophic WLP can be utilized by anaerobic microbial consortium as a carbon and energy source for reductive dehalogenation (organohalide respiration) in bioremediation of toxic substances [9,10,11].

Humic substances (HSs) are the key component of the carbon cycle on the earth, attained by virtue of biogeochemical transformation and long residence time of soil organic matter with mineral compounds [12,13,14]. Humin is understood as the insoluble fraction of HSs at any pH in the form of an organo-mineral complex, and constitutes the dominant fraction of HSs in environment [15,16]. Although only soluble form of HSs was considered to function as extracellular electron mediator (EEM) before [17,18], solid-phase HSs including humin have also been found to function as EEM [19,20,21].

The isoelectric point of humin was estimated to range from +8 mV to −300 mV (vs. standard hydrogen electrode, SHE) when measured using sodium sulfate as the electrolyte with DMSO (dimethyl sulfoxide) as the solvent; while showed redox peak at two positive potentials of +272mV and +5mV (vs. SHE) when anaerobic mineral medium was used as the solvent [19,22]. The versatility of humin thus, as EEM functioning for reducing reactions ranging from organohalide respiration [19,23]; denitrification [24]; iron(III) reduction; to dissimilatory nitrate reduction to ammonia [25], was limited to energetically favored conditions with the use of organic substrate (formate [26], lactate [27], etc.), chemical reducing agent (sodium borohydride [28]), and so on for external electron donation.

In retrospect to the interest in use of many conductive solid surfaces like modified graphene oxide [29], carbon nanotubes [30], and so forth, as the extracellular interface for electron shuttle in microbial CO_2_ reduction to acetate, little attention was given to humin as the EEM [31] despite its versatility in redox mediation and, in particular, under energy-limited conditions, a key feature distinguishing the electroautotrophic microorganisms from others [32,33].

Thus, the present study examined the effect of humin as the solid material for electron shuttle under energy-limited conditions for nonphototrophic anaerobic CO_2_ reduction to produce acetate by a mixed consortium when no external source of energy, as either organic or inorganic, was provided with sole exception to humin.

## 2. Materials and Methods 

### 2.1. Humin Extraction from Kamajima Soil

Humin was extracted from Kamajima paddy soil, Aichi prefecture, Japan, as previously reported [34]. Briefly, the sieved soil (2 mm ø pore sieving) suspended in pure water was segregated by repeated decantation of time seven seconds each. The decanted finer particles of soil were then washed alternately by the chemicals—2% hydrofluoric acid (HF) and 0.1N sodium hydroxide (NaOH)—for the elimination of clay particles, metallic ions, or humic and fulvic acids. The washing was carried out using a mechanical shaker (Taitec TS-20, Tokyo, Japan) for 24 h, followed by the centrifugation at 8000× *g* (Kubota 7780, Tokyo, Japan). The insoluble fraction obtained from soil after the washing with chemicals was neutralized to pH 7 using 0.1N hydrochloric acid (HCl), further washed with pure water to eliminate trace chloride, and finally freeze-dried. The freeze-dried sample was finely powdered using mortar and pestle for the use as humin.

### 2.2. Development of Bacterial Consortium PCPA0 and Experimental Conditions

The source inoculum was obtained from a lactate-utilizing pentachorophenol-dechlorinating consortium (LC) maintained with Kamajima soil in the laboratory. The consortium PCPA10 was developed from LC in an anaerobic mineral medium (Med-A) amended with 10 mM sodium acetate (Na-acetate) as organic electron donor, 20 µM pentachlorophenol (PCP) as the electron acceptor, and 1 g humin as redox mediator for dechlorination. Med-A was composed of ${\mathrm{NH}}_{4}\mathrm{Cl},$ 1.00 g/L; ${\mathrm{CaCl}}_{2}$·2$H_{2}O,$ 0.05 g/L; ${\mathrm{MgCl}}_{2}$·6$H_{2}O,$ 0.10 g/L; $K_{2}{\mathrm{HPO}}_{4,}$ 0.4 g/L; ${\mathrm{NaHCO}}_{3},$ 4.00 g/L; and 1 mL/L each of SL-10 trace element solution; selenite-tungstate solution; and resazurin was added [35]. The medium pH was set at 7.0 ± 0.2 by sparging with gas mixture of N_2_ and CO_2_ in ratio of 4:1. The culture vial (total volume of 120 mL) containing 50 mL of Med A was sealed with butyl rubber stopper and aluminum crimp caps. The headspace of the sealed vial was flushed with gas mixture of N_2_ and CO_2_ in ratio of 4:1 for 45 min. Sterilization was done at 121 °C for 20 min. Addition of vitamin solution [23]; 10 mM Na-acetate; 20 µM PCP (>90% purity); and reducing agent titanium nitrilotriacetic acid (Ti-NTA, 0.20 mM) was carried out prior to the inoculation. Maintenance and development of the consortia were carried out by transferring 10% (*v*/*v*) of the bacterial culture for inoculation. The cultures were incubated at 30 °C in dark for two weeks. 

Subsequently, the concentration of Na-acetate in Med-A was decreased to 1 mM, and the consortium PCPA1 was obtained. The consortium PCPA1 post third generation showed steady increase in acetate concentration higher than initial concentration of 1 mM after two weeks of incubation along with dechlorination of PCP to lower chlorinated metabolites (Figure S1, Figure S2). As such, the acetogenic consortium PCPA0 was developed from the fifth generation of PCPA1 in the same medium conditions as PCPA1, but the addition of Na-acetate as external electron donor or carbon source was omitted. 

### 2.3. Timeline Study Using Consortium PCPA0

Timeline study was carried out using sacrificing cultures of consortium PCPA0 in triplicates (for days 0, 5, 10, and 15) to examine the effects of the following conditions: (a) PCPA0-C1—absence of humin in the condition of PCPA0; (b) PCPA0-C2—absence of reducing agent in the condition of PCPA0; (c) PCPA0-C3—absence of CO_2_/HCO_3_^−^ in condition of PCPA0, that is, bicarbonate buffer was replaced with 20 mM MOPS (3-(N-morpholino)propanesulfonic acid) with nitrogen sparging, and headspace was flushed with N_2_ only. Control-1 was provided without bacterial inoculation as the basal condition of PCPA0. The inoculum source was 10th generation of the consortium PCPA0. The conditions are summarized in Table 1.

### 2.4. Effect of Carbon Monoxide (CO)

Consortium A0CO was developed from the 17th generation of PCPA0 under the same conditions of PCPA0 with exception to 3 mL addition of carbon monoxide (CO, 0.134 mmoles) into the headspace using gas-tight glass syringe (VICI, Baton Rouge, LA, USA) prior to inoculation. Batch study using consortium A0CO was carried out using the condition of PCPA0-C3 (MOPS buffer instead of bicarbonate buffer) with the addition of 3 mL of CO (the condition A0CO-C1). The A0CO-C2 was provided without addition of humin. The source of inoculum was the fourth generation of consortium A0CO. Acetate and methane productions were compared after two weeks of incubation. The conditions are summarized in Table 1.

### 2.5. Bulk Redox Potential of Med-A with Humin

A H-shaped dual chamber electrochemical cell separated with a proton exchange membrane (Nafion 117, DuPont, Japan, Tokyo) [36] was used for measurement of bulk potential of 4 g humin in 200 mL volume of anaerobic Med-A (in absence of vitamin, reducing agent, or PCP) under agitation by mechanical stirring. Coiled platinum (Pt) wires of 0.8 mm diameter and 1 m length (Nilaco, Tokyo, Japan) were used as both working, and counter electrodes; and Ag/AgCl glass electrode was used as a reference electrode (+250 ± 5 mV vs. SHE, Fusheng Analytical Instrument Co., Shanghai, China). The redox potential of the reference electrode was measured post use to check for discrepancies. The Pt wires were brushed clean, sonicated at 40 °C in pure water, and soaked in 5N HCl solution for 6 h, followed with wash in pure water before use.

### 2.6. Chemical Analyses

The measurement of chlorophenol metabolites using GCMS QP2010 (Shimadzu, Kyoto, Japan); headspace gas analysis using GC-14B gas chromatography equipped with thermal conductivity and flame ionization detectors (Shimadzu, Kyoto, Japan); and organic acid analysis using HPLC (Shimadzu LC-10AT, Kyoto, Japan) was carried out as reported previously [34]. The inorganic carbon analysis was carried out by total organic carbon analyzer (TOC-V_CPH/CPN_, Shimadzu, Kyoto, Japan). Samples for inorganic carbon analysis was filtered using membrane filter (pore size 0.22 µm), diluted in pure anaerobic water in ratio 1:15, and stored at 4 °C prior to analysis. The recovery of chlorophenols ranged from 85–101%. The chemicals used were of Special Grade (JIS) chemicals, purchased from FUJIFILM Wako Pure Chemical Corporation, Osaka, Japan.

### 2.7. Microbial Community Structure

The microbial DNA extraction was carried out from the seventh generation of consortium PCPA0 by FastDNA SPIN kit for soil (MP Biomedicals, Japan, Tokyo, Japan), and was analyzed for taxonomic classification as reported previously [34]. Briefly, Miseq next-generation sequence was carried out for the amplicons targeting V3−V4 region of 16S rRNA genes using primer sets—Pro341F (5′-CCT ACG GGN BGC ASC AG-3′) and Pro805R (5′-GAC TAC NVG GGT ATC TAA TCC-3′).

## 3. Results

### 3.1. Humin-Dependent CO_2_-Reducing Acetogenesis by Consortium PCPA0

**Figure 1:**
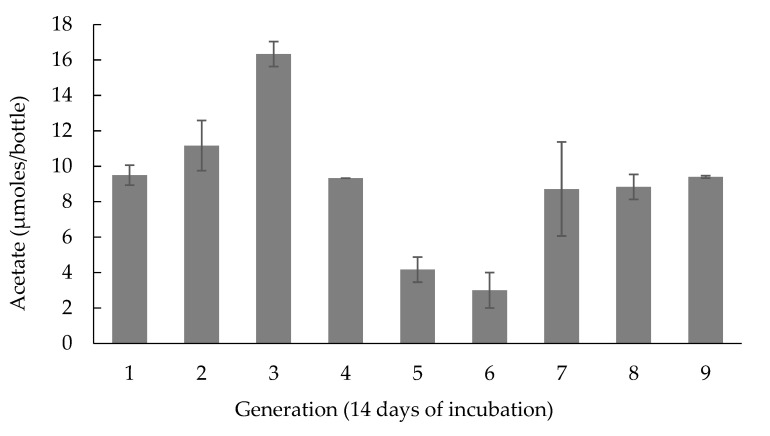
Acetate concentration observed across the generations in the consortium PCPA0. The vertical bars exhibit mean acetate amount (±standard deviation) for the triplicates after two weeks of the incubation. No external electron donor was added in the form of organic acids, or inorganic donor, or organic solvents. Nearly the same acetate concentration after two weeks of incubation across the seven generations indicated stability in the activity of the consortium PCPA0. The x-axis (*n* = 0, 1, …, 9) represents the successive generations of the consortium PCPA0.

The consortium PCPA0 was developed via acclimatization to acetate-utilizing conditions, initially to 10 mM acetate condition (the consortium PCPA10), then 1 mM acetate condition (the consortium PCPA1), and finally to the condition without acetate, as described above. Any form of external electron donor addition was omitted for the maintenance of the consortium PCPA0. Figure 1 shows the acetate concentration in the consortium PCPA0 after two weeks of incubation, across nine generations. Because 10% (*v*/*v*) transfer of the culture was carried out across the generation using the medium in absence of acetate, steady acetate concentration detected after two weeks of incubation demonstrated acetate production by the consortium PCPA0. The pH value of the culture did not change after two weeks of incubation. The production of other organic acids such as formate, lactate, or propionate was not detected. 

Additionally, anaerobic dechlorination of PCP to less chlorinated metabolites, chiefly meta-chlorophenol (3-CP) was observed (Table S1), and methane in headspace was detected as well. Hydrogen remained undetected in the headspace measurements. The results suggested acetate production without the use of any electron donor other than humin, which was investigated by timeline study on the consortium PCPA0. Based on the higher stability of acetate production after seventh generation, the microbial community structure of the consortium PCPA0 was judged to become stable, and the consortium PCPA0 post seventh generation or later was used in this study.

**Figure 2:**
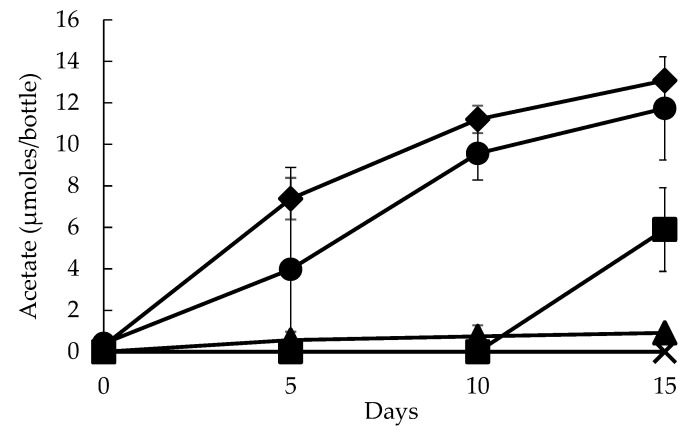
Autotrophic reductive acetogenesis by humin-dependent consortium PCPA0. The data are plotted for the self-sacrificing triplicates used for the batch culture for the timeline period of 0, 5, 10, and 15 days, except for PCPA0 where duplicates were studied. PCPA0 represents the 11th generation of consortium PCPA0 in Med-A, that is, conditioned to CO_2_/HCO_3_^−^ in medium, and headspace is flushed with N_2_/CO_2_; and humin 1g as well as 0.20 mM of reducing agent (Ti-NTA) was added to the medium (●). PCPA0-C1 had microbial source without humin in Med-A under HCO_3_^−^/CO_2_ condition (▲) with N_2_/CO_2_ in headspace. PCPA0-C2 used both humin and microbial source in Med-A, that is, under HCO_3_^−^/CO_2_ condition, but reducing agent was not added (◆). PCPA0-C3 used both bacteria and humin, but CO_2_/HCO_3_^−^ was substituted with MOPS in Med-A, and headspace was flushed with N_2_ only (■); Control-1 used only humin in Med-A (**×**) under N_2_/CO_2_ atmosphere without bacteria. The acetate values represent the mean with standard deviation shown by vertical lines.

Figure 2 shows a timeline of reductive acetogenesis by the consortium PCPA0 using humin. A gradual increase in acetate for the consortium PCPA0 was observed, reaching about 12 µmoles/bottle in the period of 15 days of incubation. In contrast, no acetate production was witnessed in Control-1, the condition without microbial inoculum, confirming that acetate production was due to microbial activity only. Also, no hydrogen or methane production was detected for Control-1. There was little to no increase in acetate for the condition PCPA0-C1 without humin. On the other hand, a steady increase in acetate was observed for the condition PCPA0-C2 without reducing agent, but using humin. The results demonstrated that while reducing agent as Ti-NTA had no pronounced effect in acetate production, humin was critical. In the condition PCPA0-C3 (without CO_2_/HCO_3_^−^), acetate production was retarded by 10 days. This retardation suggested that CO_2_/HCO_3_^−^ was utilized mainly as inorganic carbon source for the acetate production in the consortium PCPA0, and the organic fraction of humin was not the major carbon source for acetate production. The humin dependency in acetate production by the consortium PCPA0 without any other external input of electron donor suggested that humin donated electrons to the microorganisms for CO_2_ reduction, by functioning as EEM rather than as the organic matter. The electron donation by humin was also observed in the reduction of Med-A with humin, where the bulk redox potential of Med-A measured at −80.0 mV (vs. Ag/AgCl) with suspended humin (4 g/L) at pH 6.8, while it measured at +42.6 mV (vs. Ag/AgCl) without humin. Further analysis for the condition PCPA0-C3 suggested that the retarded acetate production would be attributed to the CO_2_ carryover by 10% (*v*/*v*) culture transfer for the inoculation (Figure S3).

### 3.2. WLP in the Acetogenesis by Consortium PCPA0

Reductive acetogenesis using CO_2_ as the terminal electron acceptor suggested the presence of WLP in the consortium PCPA0. The PCR targeting the gene encoding formyltetrahydrofolate synthetase (FTHFS) [37], a key enzyme in WLP, gave the amplified products around 1.1 kilo base pairs (Figure S4), supporting the occurrence of WLP in the consortium. 

**Figure 3:**
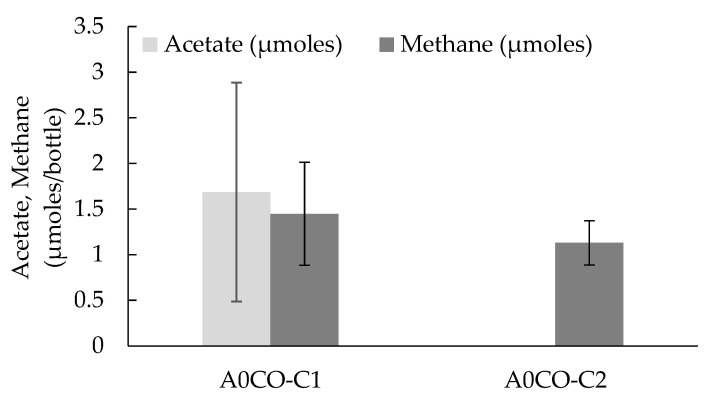
Acetate production and methane generation for conditions A0CO-C1 and A0CO-C2. The condition A0CO-C1 was studied using 1g humin/bottle, whereas no humin was added to the condition A0CO-C2. The vertical bars are representative data of the triplicates studied for two weeks incubation, with the vertical lines as the standard deviation.

Humin dependency for the electron donation was also witnessed in the conversion of CO to acetate in the consortium A0CO. CO is the substrate of carbon monoxide dehydrogenase/acetyl-CoA synthase, an essential enzymatic complex for WLP, although CO also functions as the reducing agent. The condition A0CO-C1 with humin showed acetate production; whereas no acetate was detected at all for A0CO-C2 without humin (Figure 3). The results are consistent with the involvement of WLP in the acetogenesis by consortium PCPA0. On the other hand, methane production was observed in the conditions of both A0CO-C1 and A0CO-C2. Methane production remained unaffected by the presence of humin. The methanogenesis observed would be supported by the reducing power of CO as the inorganic electron donor ($E^{o^{\prime }},\;\mathrm{CO}/{\mathrm{CO}}_{2}=-520\;\mathrm{mV}$) [38].

### 3.3. Microbial Community Structure of Consortium PCPA0

**Figure 4:**
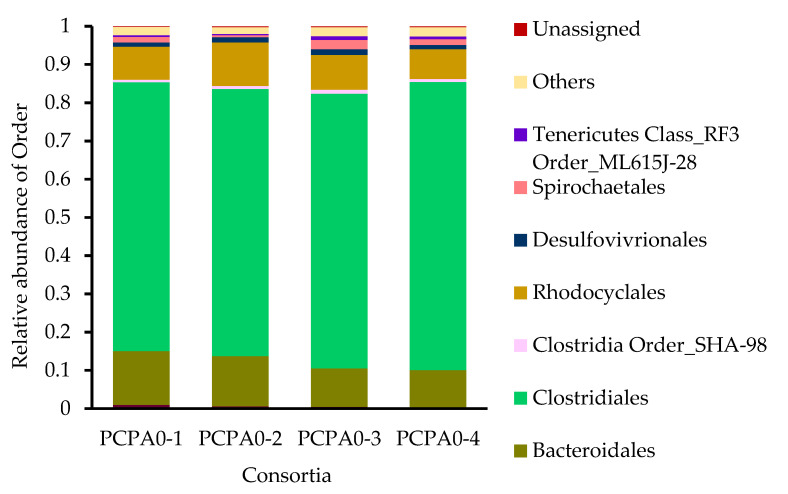
Community structure of consortium PCPA0 based on 16S rRNA gene sequencing. The data shows the community structures of four replicates of the consortium PCPA0, individually. “Others” denotes the populations less than 0.5% abundance. “Unassigned” represents the genes that were not assigned to any taxonomic position by blast sequencing.

Figure 4 represents the microbial community structure of consortium PCPA0 at the order level. The bacteria classified into the order *Clostridiales* (*Firmicutes*) occupied 71.8 ± 2.5% of total population. Other major groups belonged to *Bacteroidales* (*Bacteroidetes*, 11.8 ± 2.2%), *Rhodocyclales* (β-*Proteobacteria*, 9.2 ± 1.5%), *Desulfovibrionales* (δ-*Proteobacteria*, 1.3 ± 0.2%), and *Spirochaetales* (*Spirochaetes*, 1.5 ± 0.8%). The microbial community structure was in accordance with the CO_2_-reducing acetogenesis because WLP has been observed mostly in *Firmicutes*, and some in δ-*Proteobacteria* and *Spirochaetes* [8,38]. *Lachnospiraceae* (21.6 ± 1.3%) and *Ruminococcaceae* (16.4 ± 0.7%), two major family in *Clostridiales* detected, have been reported as CO_2_-reducing acetogens [39]. *Dehalobacter* occupying 3.7 ± 0.8% of total abundance was considered as the PCP-dechlorinating bacterium, utilizing reduced humin as the electron donor [19]. The CH_4_ production was attributed to *Methanobacteriaceae* (*Euryarchaeota*, <0.35% abundance), CO_2_-reducing methanogen with WLP [40].

### 3.4. Electron Donating Capacity of Humin

It is understood that acetogenesis through WLP is not constrained by use of the organic substrates, hydrogen, or any other electron donors, but only requires production of acetate through CO_2_ reduction [8,38]. In this study, the acetogenic consortium PCPA0 was considered to utilize electrons from humin as the electron mediator. Methanogenesis and anaerobic PCP dechlorination observed in the consortium PCPA0 would utilize the electrons from humin. Table 2 shows the estimation of electron-donating capacity of humin based on the amount of acetate produced, as well as from the amount of methane due to methanogenesis, and chlorophenol metabolites in anaerobic PCP dechlorination based on the following equations [41,42,43].

(1) $$2{\mathrm{CO}}_{2}+4H_{2}↔{\mathrm{CH}}_{3}\mathrm{COOH}+2H_{2}O$$

(2) $${\mathrm{CO}}_{2}+4H_{2}\to {\mathrm{CH}}_{4}+2H_{2}O$$

(3) $$C_{6}{\mathrm{Cl}}_{5-x}H_{x}\mathrm{OH}+H2_{2}\to C_{6}{\mathrm{Cl}}_{4-x}H_{x+1}\mathrm{OH}+\mathrm{HCl},\;\left(x=0-5\right)$$

In the equations above, hydrogen is shown as the reducing equivalent, where one hydrogen atom equals to one electron plus one proton. The production of one molecule of acetic acid from CO_2_ requires eight reducing equivalents. Again, the production of methane requires eight reducing equivalents, and the anaerobic dechlorination of one chlorine atom requires two reducing equivalents. In Table 2, the difference in the amounts of acetate, methane, and chlorophenols between PCPA0 and PCPA0-C1 indicated the tendency of electron donation of humin to the respective group of microorganisms. Mass balance study of the chlorophenols indicated possible decomposition of phenol structure in consortium PCPA0, as the unaccounted chlorophenols. The unaccounted chlorophenols were estimated to range from zero to thirty percent. In consideration with benzoyl-CoA pathway [44] for phenol decomposition, the acetate production from chlorophenols was estimated. The results showed that the possible contribution of chlorophenols was not more than 3 µmols/bottle (27 %) for acetate production. The total electron-donating capacity of humin was estimated to be larger than 87 µeeq/g humin for the consortium PCPA0, where electron donation towards acetate production specifically was larger than 62 µeeq/g humin in consideration with the other biochemical pathways. This suggested that methane decomposition or phenol decomposition did not contribute as secondary influences for the acetate production observed in the consortium PCPA0.

## 4. Discussion

It was demonstrated for the first time that humin supported microbial CO_2_-reducing acetogenesis by donating electrons as EEM to the consortium PCPA0. The comparison of acetate production in the consortium PCPA0 under the different conditions clearly showed that acetate was not produced from organic fraction of humin but from CO_2_ (Figure 2). The consortium PCPA0, predominated by *Clostridiales* (Figure 4), was maintained with humin as EEM for sole source of electrons with the presence of CO_2_/HCO_3_^-^ as carbon source over the generations (Figure 1), suggesting the occurrence of microbial growth under the energy-limited conditions. Humin also supported dechlorination and methanogenesis of the consortium PCPA0 (Table S1). These suggested that under energy-limited conditions, humin served electrons as a solid-phase EEM for the multiple reactions by microorganisms including electroautotrophs.

**Figure 5:**
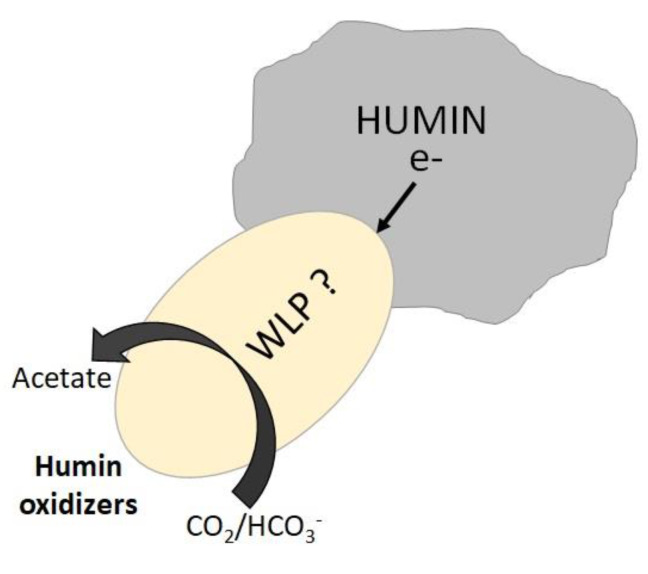
Microbial CO_2_-reducing acetogenesis supported by humin as EEM for the electron donation to the consortium PCPA0 in the CO_2_/HCO_3_^-^-buffered medium.

Supporting microbial CO_2_-reducing acetogenesis by humin as EEM suggested that humin can donate electrons for highly reduced reactions, because microbial CO_2_-reducing acetogenesis involves complex enzymes which require more reducing conditions than −290 mV ($E^{o^{\prime }}$) [43]. Previous study [25] showed the electron transfer from humin to the microorganisms implementing reductive dechlorination ($E^{o^{\prime }},$ higher than +300 mV) [41]; denitrification ($E^{o^{\prime }}$, higher than +400 mV), iron reduction ($E^{o^{\prime }},$ about 0 mV), and dissimilatory nitrate reduction to ammonia ($E^{o^{\prime }},$ about +360 mV) [45]. It was also previously suggested the redox potential of humin ranging from +272 mV to −300 mV (vs. SHE) as redox mediator [19,22,25], indicating that the electron transfer from humin was favorable. However, for the CO_2_-reducing acetogenesis, the electron donation from humin would not be expected based on the redox potential of humin. In a recent study it was argued that a higher redox potential of −340 mV (vs. SHE) could induce such reductive acetogenesis in *Acetobacterium woodii* [46]. It is understood that the organic chemical structures of HSs change three dimensionally depending on the environmental conditions [47]. Therefore, it can only be proposed that the various domains with different redox potentials are present in organic fractions of humin, and the highly reduced domains storing electrons were accessible to the humin-dependent acetogenic community present in the consortium PCPA0 for electron harvest, as shown in Figure 5.

In comparison to the redox potential of half reaction of CO_2_/CH_3_COOH at −290 mV $\left(E^{o^{\prime }}\right)$, the redox potential of CO_2_/CH_4_ at −230 mV $\left(E^{o^{\prime }}\right)$ [42], and those in the series of PCP dechlorination to phenol which range from +333 mV to +424 mV $\left(E^{o^{\prime }}\right)$ [41] are higher. As the preference of electron-accepting reactions at higher redox potentials comes first, before those at lower redox potentials, the reducing reactions should occur in the order of anaerobic PCP dechlorination, methanogenesis, and lastly, acetogenesis. This explained the result that most of the PCP was dechlorinated in the consortium PCPA0, but not the result that acetogenesis was dominant over methanogenesis. This could be attributed only to the predominance of acetogenic microorganisms in the consortium. 

The consortium PCPA0 with humin in the CO_2_/HCO_3_^−^-buffered dechlorinating medium did not produce longer-chain organic acids, and even lactate, while acetate production was witnessed. This indicated energy-limited conditions, as production of longer-chain fatty acids requires more energy [33]. The detection of formate was expected [5], but not in the consortium PCPA0 with humin where WLP was considered to be involved in the autotrophic acetogenesis, as indicated by the acetogenesis from CO (Figure 3) and the presence of FTHFS genes (Figure S4). The electron transfer between humin and WLP for acetate-producing microorganisms hints at complex biochemical interaction (Figure 5), and further study is required to explore the mechanism of EEM function of humin. Although *Lachnospiraceae* and *Ruminococcaceae* were detected as major *Clostridiales* in the consortium PCPA0 (Figure 4), the possible involvement of other bacteria cannot be discarded. Thus, the identification of microorganisms responsible for the humin-dependent acetogenesis would be critical for elucidation of the mechanism. 

## 5. Conclusions

This study demonstrated that humin can serve extracellular electrons to microbial CO_2_-reducing acetogenesis, requiring highly reduced redox potential at −290 mV $(E^{o^{\prime }}$), under energy-limited dark conditions. This distinctive feature of humin was successfully used by the anaerobic consortium PCPA0 for autotrophic acetate production (11 µmoles/g-humin) in absence of any degradable organic compounds, hydrogen, or electricity. The major group of bacteria in the consortium PCPA0 belonged to the order *Clostridiales*, followed by *Bacteroidales*, *Rhodocyclales*, and *Desulfovibrionales*. Reductive dechlorination and methanogenesis observed in the consortium PCPA0 were also supported by humin as electron donor. The energetics in the anaerobic consortium PCPA0 suggested that highly reduced domain occluded in humin could be accessed by certain group of microorganisms for electron harvest. Further study should be carried out to elucidate the mechanism of EEM function of humin, especially the electron-donating mechanism for microbial CO_2_-reducing acetogenesis under the conditions exerted by the consortium PCPA0. Considering the ubiquitous presence of humin in the environment, the findings significantly contribute in understanding the microbial reactions involved in the carbon cycle on the earth, and open scope to study the flexibility of using humin as the sole source of energy for bioremediation.

## Figures and Tables

**Table 1:** Experimental setup in this study. The notation “+” and “−” indicates the presence and absence of the respective conditions.

Conditions	Humin	Ti-NTA	Buffer	Headspace Composition	Inoculum Source
PCPA0	+	+	CO_2_/HCO_3_^−^	N_2_/CO_2_	PCPA0
PCPA0-C1	−	+	CO_2_/HCO_3_^−^	N_2_/CO_2_	PCPA0
PCPA0-C2	+	−	CO_2_/HCO_3_^−^	N_2_/CO_2_	PCPA0
PCPA0-C3	+	+	MOPS	N_2_	PCPA0
Control-1	+	+	CO_2_/HCO_3_^−^	N_2_/CO_2_	-
A0CO	+	+	CO_2_/HCO_3_^−^	N_2_/CO_2_/CO	PCPA0
A0CO-C1	+	+	MOPS	N_2_/CO	A0CO
A0CO-C2	−	+	MOPS	N_2_/CO	A0CO

Ti-NTA is the reducing agent used for the anaerobic medium preparation. The term PCPA0 denotes a PCP dechlorinating culture using 0mM acetate concentration.

**Table 2:** Metabolites produced by the consortium PCPA0 and the estimated electron-donating capacity of humin (1g/bottle).

Observed Reactions	Experimental Conditions		Difference Made by Humin Addition	Electron-Donating Capacity of Humin
	PCPA0 µmoles/Bottle (Mean ± SD)	PCPA0-C1 µmoles/Bottle (Mean ± SD)	µmoles/g-Humin (Mean ± SD)	µeeq/g-Humin (Mean ± SD)
Acetate Production	11.75 ± 1.75	0.91 ± 0.04	10.80 ± 1.75	86.4 ± 14.0
Methane Production	2.25 ± 0.92	n.d. ^1^	2.25 ± 0.92	18.0 ± 7.36
Total Dechlorination	4.03 ± 0.25	0.97±0.48	3.06 ± 0.73	6.12 ± 1.46
Unaccounted Chlorophenol ^2^	0.97 ± 0.25	n.d.	(2.91 ± 0.75) ^3^	(23.3 ± 6.0) ^3^
Total capacity of electron donation from humin	-	-	-	87.2

The table compares the electron-donating capacity of the consortium PCPA0 with that of the condition PCPA0-C1 without humin studied in the same timeline. The data is representative of the triplicates mean and standard deviation (SD) post fifteen days of incubation. The electron-donating capacity as electron equivalent (eeq) of 1 g humin per bottle has been calculated based on the reducing equivalents per equation 1−3. ^1^ “n.d.” denotes “not detected”. ^2^ “Unaccounted chlorophenols” denotes the unaccounted chlorophenols based on the mass balance of chlorophenols in the bottle. ^3^ The equivalent contribution as acetate production from phenol has been calculated based on benzyl-CoA pathway [44], and the equivalent contribution as electrons was subtracted from the estimated electron-donating capacity of humin.

## Supplementary Materials

The following are available online at https://www.mdpi.com/1660-4601/17/12/4211/s1, Table S1: Dechlorination metabolites detected in the timeline study using consortium PCPA0; Figure S1: Trend of acetate in consortium PCPA1 after two weeks incubation; Figure S2: Trend of dechlorination metabolites in consortium PCPA1 after two weeks incubation; Figure S3: Inorganic carbon as carbon dioxide in the mineral medium and headspace under the condition of PCPA0-C3; Figure S4: PCR products of consortium PCPA0 using specific primer set targeting the gene encoding formyltetrahydrofolate synthetase (FTHFS).
