# Supplementary material for: Humin Assists Reductive Acetogenesis in Absence of Other External Electron Donor
Source: Int J Environ Res Public Health. 2020 Jun 12;17(12):4211. doi: 10.3390/ijerph17124211 (PMC7344539; doi:10.3390/ijerph17124211)
Supplement: Supplementary file 1 [file ijerph-17-04211-s001.pdf]

Supplemental Information for

Humin Assists Reductive Acetogenesis in Absence of Other External Electron Donor

Mahasweta Laskar [1,2], Takuya Kasai [1,2], Takanori Awata [3] and Arata Katayama [1,2,*]

[1] Graduate School of Engineering, Nagoya University, Nagoya 464-8603 Japan
[2] Institute of Materials and Systems for Sustainability, Nagoya University, Nagoya 464-8603 Japan
[3] National Institute for Land and Infrastructure Management, Tsukuba 305-0804 Japan
* Correspondence: katayama.arata@nagoya-u.jp; Tel.: +81-(0)52-789-5856

Number of Pages: 4
Number of Figures: 4
Number of Tables: 1

Table S1. Dechlorination metabolites detected in the timeline study using consortium PCPA0. The table presents the different chlorinated metabolites observed at Day 15 for the consortium PCPA0, and the other conditions studied. The notation (o) indicates the detection of the respective metabolite, while the notation (x) indicates that the respective metabolite was not detected.

Metabolites at Day 15	Experimental Conditions			
	PCPA0	PCPA0-C1	PCPA0-C2	PCPA0-C3
PCP	o	o	x	x
2,3,4,5-tetrachlorophenol	o	x	x	x
3,4,5-trichlorophenol	x	x	x	x
3,5-dichlorophenol	o	x	x	x
3-chlorophenol	o	x	o	o
phenol	x	x	x	x

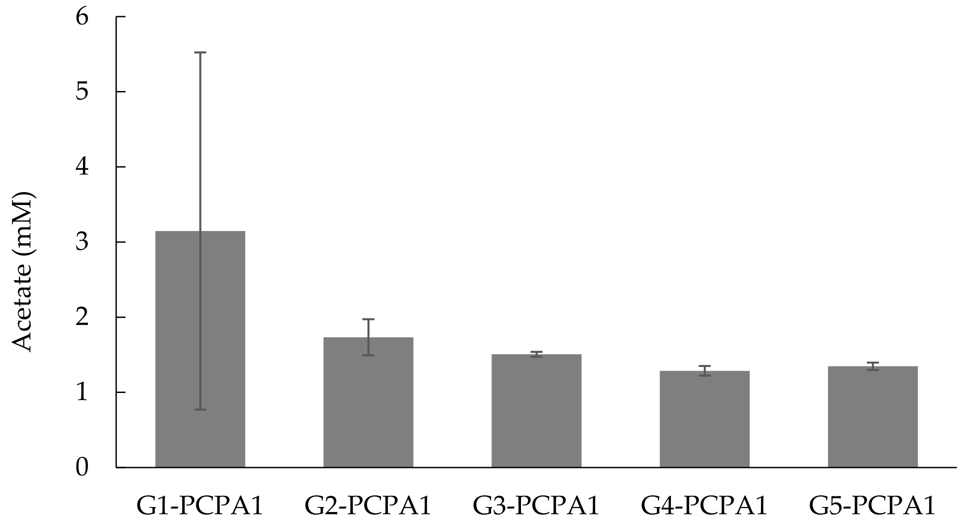

Figure S1. Trend of acetate in consortium PCPA1 after two weeks incubation. The vertical bars represents the amount of acetate (mM) detected for consortium PCPA1 in triplicates with the vertical lines as the standard deviation post two weeks incubation. The consortium PCPA1 was amended with 1mM acetate on Day 0. The x-axis represents the successive generations, the notation ‘G(n)’ indicating the respective generation.

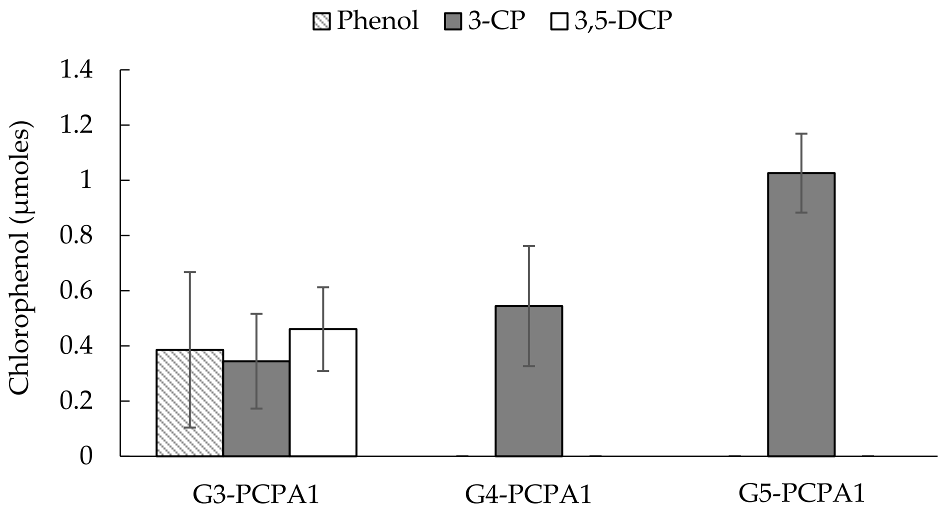

Figure S2. Trend of dechlorination metabolites in consortium PCPA1 after two weeks incubation. The vertical bars represents the amount of chlorophenols (µmoles) detected for consortium PCPA1 in triplicates with the vertical lines as the standard deviation post two weeks incubation. The consortium PCPA1 was amended with 1mM acetate on Day 0, and 20µM pentachlorophenol was provided as the electron acceptor, i.e.,1µmoles as PCP. The x-axis represents the successive generations, and the notation 'G(n)' indicating the respective generation. The legend 3-CP and 3,5-DCP indicates meta-chlorophenol, and 3,5-dichlorophenol.

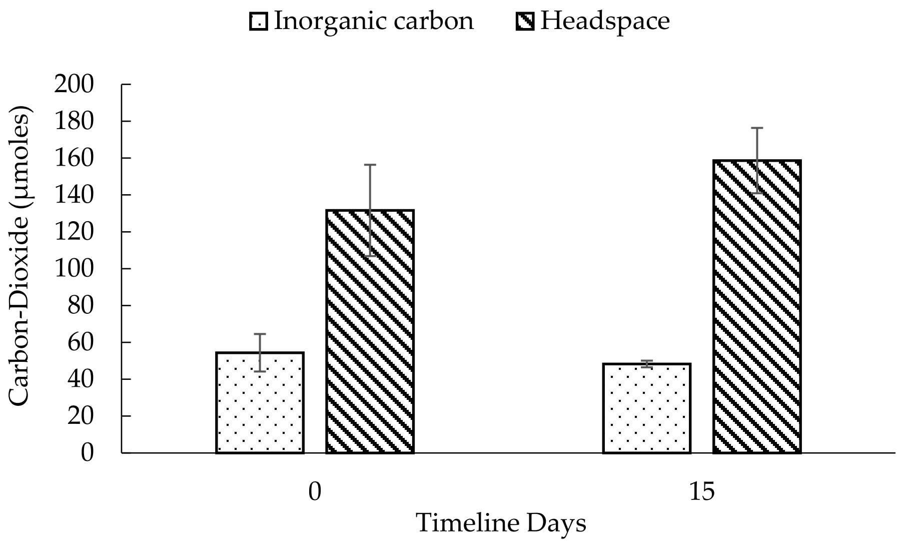

Figure S3. Inorganic carbon as carbon-dioxide in the mineral medium and headspace under the condition of PCPA0-C3. The figure represents the data for self-sacrificing triplicates for day 0 and day 15. The vertical lines are standard deviation of the triplicates. The carbon dioxide was present because of carry over by 10% inoculation in the condition PCPA0-C3, as the medium and headspace did not contain carbon dioxide prior to the inoculation.

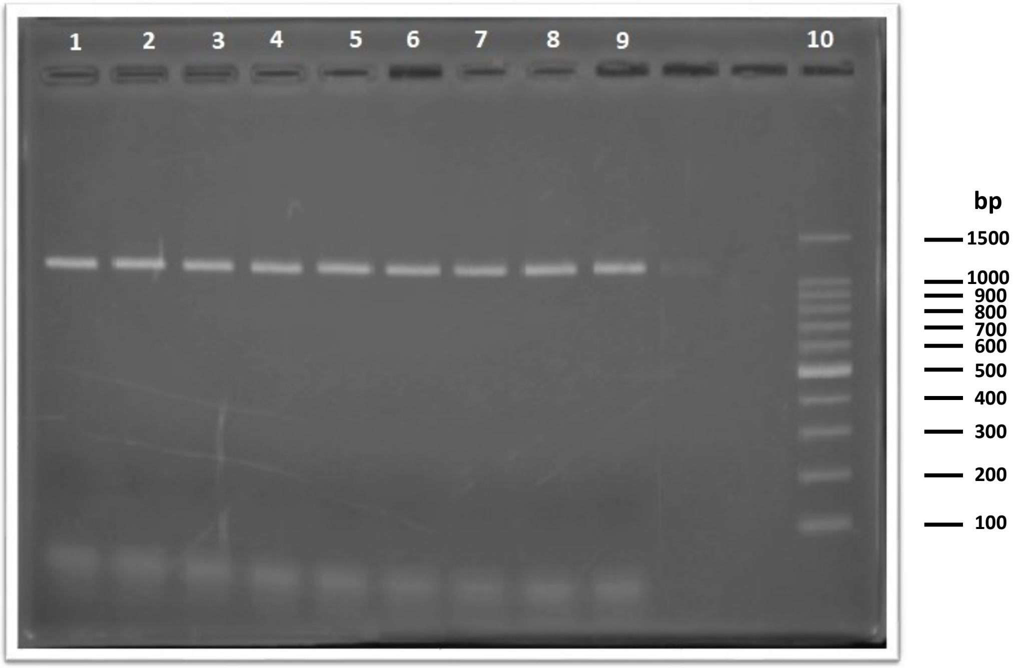

Figure S4. PCR products of consortium PCPA0 using specific primer set targeting gene encoding formyltetrahydrofolate synthetase (FTHFS). The bands detected are amplified PCR products for the consortium PCPA0 post two weeks of incubation. The lanes marked 1 till 9 are extracted from the replicates belonging to the 11th generation of the consortium PCPA0 where lane 1-3, 4-6, and 7-9 are in triplicate for each of the respective replicates. The lane 10 represents the 100 base pair (bp) DNA ladder (TaKaRa Bio, Kusatsu, Shiga, Japan). The primers-FTHFSf (5′ TTYACWGGHGAYTTCCATGC-3′) and FTHFSr (3′-GTATTGDGTYTTRGCCATACA-3′) were used to target the gene of tetrahydrofolate synthetase (FTHFS), and used the PCR protocol for amplification at 53°C for 30 cycles as described in the literature [1]. The 20µL PCR mix used- 5µL DNA; 2µL 10X Tae Buffer; 2µL dNTP mix; 0.5µL each of FTHFSf and FTHFSr primers; 0.1µL Ex Taq Polymerase; and 9.9µL pure water. For product identification, 1% agarose gel was used with 100bp DNA ladder as marker.

References

1. Henderson, G.; Naylor, G.E.; Leahy, S. C.; Janssen, P. H. Presence of novel, potentially homoacetogenic bacteria in the rumen as determined by analysis of formyltetrahydrofolate synthetase sequences from ruminants. *Applied and Environmental Microbiology* **2010**, *76* (7), 2058–2066. https://doi.org/10.1128/AEM.02580-09.
